# An Assessment of Engineered Calcium Oxalate Crystal Formation on Plant Growth and Development as a Step toward Evaluating Its Use to Enhance Plant Defense

**DOI:** 10.1371/journal.pone.0141982

**Published:** 2015-10-30

**Authors:** Paul A. Nakata

**Affiliations:** USDA/ARS Children’s Nutrition Research Center, Department of Pediatrics, Baylor College of Medicine, Houston, TX United States of America; Ghent University, BELGIUM

## Abstract

The establishment of new approaches to control chewing insects has been sought not only for direct use in reducing crop loss but also in managing resistance to the pesticides already in use. Engineered formation of calcium oxalate crystals is a potential strategy that could be developed to fulfill both these needs. As a step toward this development, this study investigates the effects of transforming a non-calcium oxalate crystal accumulating plant, *Arabidopsis thaliana*, into a crystal accumulating plant. Calcium oxalate crystal accumulating *A*. *thaliana* lines were generated by ectopic expression of a single bacterial gene encoding an oxalic acid biosynthetic enzyme. Biochemical and cellular studies suggested that the engineered *A*. *thaliana* lines formed crystals of calcium oxalate in a manner similar to naturally occurring crystal accumulating plants. The amount of calcium oxalate accumulated in leaves also reached levels similar to those measured in the leaves of *Medicago truncatula* in which the crystals are known to play a defensive role. Visual inspection of the different engineered lines, however, suggested a phenotypic consequence on plant growth and development with higher calcium oxalate concentrations. The restoration of a near wild-type plant phenotype through an enzymatic reduction of tissue oxalate supported this observation. Overall, this study is a first to provide initial insight into the potential consequences of engineering calcium oxalate crystal formation in non-crystal accumulating plants.

## Introduction

Plants utilize different strategies to protect themselves against herbivory (reviewed by [1–4]). These strategies include biochemical and physical mechanisms which can be either formed during plant growth and development or inducible by feeding. Phytochemicals deter herbivory by acting as antifeedants, toxins, or by interfering with digestion. Structural defenses such as leaf hairs, thorns, or thick cuticles serve to protect by acting as a physical barrier to feeding. Oxalic acid is one compound that functions as part of both biochemical and physical mechanisms of defense depending upon the form of the acid present in the plant tissue (reviewed by [5–8]).

In the soluble form, oxalate has been shown to act as a biochemical deterrent. The free acid acts as a toxin and can have lethal consequences when ingested in large quantities. This has been shown to be a problem for grazing animals especially when their diet is restricted to consumption of plant foods high in soluble oxalate as a result of environmental factors such as drought [9]. Deaths of entire flocks of free grazing sheep have been reported [9]. Soluble oxalate has also been shown to act as a chemical deterrent to smaller sucking insects such as plant hoppers [10, 11] where the insects were observed to abandon the oxalic acid containing resistant varieties after secretion of saliva or honey dew. It has been proposed that oxalic acid is part of a defensive mechanism that inhibits insect sheath building [12]. The acid has also been shown to exert inhibitory effects on aphids [13].

In the insoluble form, oxalate is bound with calcium to form the calcium oxalate crystal. These crystals have been shown to act as a physical deterrent [6–8, 13]. The plant *Tragia ramosa*, for example, is covered with stinging hairs. Each stinging hair consists of an elongated cell which encompasses a large needle-shaped styloid crystal(s) made of calcium oxalate [14]. When an animal comes into contact with this plant, the tip of the elongated cell ruptures exposing the needle-shaped crystal which can puncture the dermis of the animal. A toxin is then channeled from the base of the cell along a groove that runs along one edge of the needle-shaped crystal. It is this toxin which then causes the stinging sensation [14]. Needle-shaped crystals in the leaves and corms of edible aroid species have been known to cause swelling of the mouth and throat of people consuming these plants [15]. Similar crystals have been reported to cause contact dermatitis in the workers farming flowers such as daffodils [16] and growers of agave [17]. One can easily envision that large needle shaped crystals can act as a deterrent against larger animals, but the crystals do not have to be that large or needle-shaped to deter smaller targets such as chewing insects. Studies [18, 19] utilizing the model legume, *Medicago truncatula* showed that even the smaller non-needle shaped prismatic crystals can act as a deterrent against chewing caterpillar larvae such as the beet armyworm, *Spodoptera exigua*. These smaller prismatic crystals act as a physical abrasive that causes damage to the caterpillar mandibles (teeth) during feeding [18, 19].

Although calcium oxalate is common to plants, a number of species including some important crop plants (e.g., canola, maize) do not accumulate appreciable amounts of calcium oxalate crystals. Thus engineering such non-crystal forming plants to accumulate crystals of calcium oxalate is a potential novel strategy to increase resistance to chewing pest without the need of additional chemical pesticides. Recently a study revealed the possibility of transforming *Arabidopsis*, a non-crystal forming plant, into a crystal forming plant [20]. The engineered plants appeared to form calcium oxalate in a manner similar to natural crystal forming plants indicating that the components of the basic calcium oxalate formation machinery may be functionally conserved even in non-crystal accumulating plants. Although this finding raised the probability of engineering calcium oxalate crystal formation in non-crystal forming plants as a viable strategy to increase resistance to chewing insects the amount of calcium oxalate accumulated was several-fold lower than the amounts accumulated by plants such as *M*. *truncatula* where crystals have been shown to play a defensive role. Therefore, it is likely that higher calcium oxalate concentrations will need to be achieved before rational strategies can be designed to engineer crystal formation for plant protection. The desire to increase calcium oxalate concentrations raises a number of questions. Such questions include what is the viability of a plant engineered to form crystals of calcium oxalate? How much calcium oxalate can a plant be engineered to accumulate? How much calcium oxalate can a plant tolerate? What tissues can be transformed to produce such crystals? Are there detrimental effects on growth and development?

As a step toward answering these questions, this study reports the generation and characterization of *Arabidopsis* lines that have been engineered to accumulate higher amounts of calcium oxalate crystals. The higher amount of crystal formation was achieved by expressing a single oxalate biosynthetic gene, obc1, from the oxalate-secreting animal pathogen, *Burkholderia mallei* [21] in *Arabidopsis*. Through phenotypic and biochemical analysis of this plant line the effects of engineering calcium oxalate crystal accumulation in a non-crystal accumulating plant were assessed. The generation of an obc1 double mutant through retransformation with an oxalyl-CoA synthetase [22, 23] expression construct aided in assigning the phenotypic and biochemical findings to the increase in calcium oxalate accumulation.

## Materials and Methods

### Generation of obc1 expression construct

The obc1 pDUET expression construct [21] was digested with *Nde*I and the site filled in using klenow (New England Biolabs, Inc., Ipswich, MA) according to manufacturer’s instructions. The obc1 ORF was then liberated by digestion with *Kpn*I and gel purified using the Wizard SV gel and PCR clean up system (Promega, Madison, WI). The purified fragment then was ligated into the pBin19 plant gene expression vector that had been digested with SmaI-KpnI. DNA sequencing was conducted to confirm the proper orientation of the ORF in the pBin19 construct. The obc1 plant expression construct was transformed into *Agrobacterium tumefaciens* strain GV3101.

### Generation of oxalyl-CoA synthetase expression construct

RNA was isolated from leaves of 4 week old *M*. *truncatula* using Tripure reagent (Roche) according to the manufacturer’s instructions. Total RNA was used for first strand cDNA synthesis using oligo dT and Superscript III first strand synthesis supermix (Invitrogen, Carlsbad, CA). Four microliters of each reverse transcription reaction then was amplified by PCR using the AccuPrime^TM^ DNA polymerase kit (Invitrogen) according to manufacturer’s recommendations. All hybridization steps were performed using a MJ Research PTC-2 thermal cycler (Biorad, Hercules, CA) with the following parameters: 95°C for 3 min, 30 cycles of 95°C for 30 sec, 55°C for 30 sec, and 72°C for 1 min. After completion of the 30 cycles, a 10 min extension was run at 72°C. The gene specific primers 5’-ACCCGGGATGGAAACCGCTACAACCCTC-3’ and 5’-AGAGCTCTCAAGCTTGAGAGACAAAGTG-3’ for the oxalyl-CoA synthetase contained a SmaI and SacI site on the N-terminal and C-terminal primers, respectively. The amplified fragment was cloned using the Qiagen TA cloning kit (Qiagen Inc., Valencia, CA). This construct was digested with SmaI-SacI and the fragment encoding the *M*. *truncatula* oxalyl-CoA synthetase (MtAAE3) isolated by agarose gel electrophoresis and purified using the Wizard SV Gel and PCR Clean-up System (Promega). The purified MtAAE3 fragment then was ligated into the complementary SmaI-SacI sites of the pCAMBIA3300 with the β-glucuronidase (GUS) gene excised. The resulting construct was transformed into *A*. *tumefaciens* strain GV3101.

### Generation of transgenic plants

*Arabidopsis thaliana* ecotype Columbia was transformed using the floral dip method [24]. Seeds from the transformed plants were selected on Gamborg B-5 basal media (PhytoTechnology Laboratories, Shawnee Mission, KS) plates containing 25 μg ml^-1^ kanamycin (A.G. Scientific, Inc., San Diego, CA) or a combination of 25 μg ml^-1^ kanamycin and 10 μg ml^-1^ glufosinate-ammonium (Sigma-Aldrich, St. Louis, MO) to select for the obc1 and obc1 + oxalyl-CoA synthetase transgenic plants, respectively. The selected plants were then transferred to soil, self-pollinated, and the seeds collected. The generation of the obcAobcB transgenic plant was described previously [20]. Control plants were generated by transforming wild-type *Arabidopsis* with the empty pBin19 vector.

### Plant growth

Wild-type and transgenic seeds were surface-sterilized by immersion in 70% (v/v) ethanol for 5 min, followed by a 10 min immersion in 10% bleach solution, and then rinsed 4–5 times with sterile water. The sterilized seeds then were germinated on 1X Gamborgs B-5 basal media pH 5.7 containing 0.5% (w/v) sucrose and solidified with 0.7% (w/v) agar. Plates were placed at 4°C for 3 days and then germinated under 150 μE of continuous light in a 22°C growth chamber (Percival Scientific, Perry, IA). Germinated plants then were transferred to pots containing Sunshine Professional Growing mix soil (Sungro Horticulture, Agawam, MA) and grown under 100 μE (16h day/8 h night) at 24°C.

### Gene expression

RNA was isolated from leaves of 3–4 week old *Arabidopsis* using Tripure reagent (Roche) according to the manufacturer’s instructions. Total RNA was used for first strand cDNA synthesis using oligo dT and Superscript III first strand synthesis supermix (Invitrogen). Four microliters of each reverse transcription reaction then was amplified by PCR using the AccuPrime^TM^ GC-rich DNA polymerase kit (Invitrogen) according to manufacturer’s recommendations. All hybridization steps were performed using a PTC-2 thermal cycler (MJ Research) with the following parameters: 95°C for 3 min, 30 cycles of 95°C for 30 sec, 62°C for 30 sec, and 72°C for 1 min. After completion of the 30 cycles, a 10 min extension was run at 72°C. The gene specific primer sets used were 5’-CTGGCGAACGGGATGGC-3’ and 5’-GCATGATCGTCGAGCAGGAAC-3’ for obc1; CGTGGTGACGGTCGGCTC-3’ and 5’-CTGGGGGTCGCGCTCG-3’ for obcA; GACGCGTTCGTGGCCG-3’ and 5’-CGTCACGCGTACCAGCTCG-3’ for obcB; 5’-GAGTCAACACAATTTAGTTTCATCGGTTCG-3’ and 5’- CTTGCAATATTTTAGCACCTCTTCAGCATC-3’ for *M*. *truncatula* oxalyl-CoA synthetase; and 5’-CAGAAAGATGCTTACGTTGGTGATGAAG-3’ and 5’-CAAACTCACCACCACGAACCAGATAA- 3’ for actin 2 (control).

### Leaf clearings

Leaf samples were harvested from 4 week old plants and cleared in 95% (v/v) ethanol. The leaf samples then were equilibrated with water and visually inspected for calcium oxalate crystal deposition using light microscopy and crossed-polarizers. Images of whole-leaf mounts were captured using a CCD72 camera mounted on a Zeiss Axiophot light microscope.

### Microscopy

Leaf tissue was dissected into 2 mm^2^ pieces and fixed in 2% paraformaldehyde/2% glutaraldehyde in 100 mM PIPES (piperazine-N,N’-bis[2-ethanesulfonic acid]) pH 7.2 for 2 days at 4°C. The samples were postfixed in 1% phosphate buffered osmium tetroxide. Samples then were dehydrated in a graded ethanol series, washed twice with polyprylene oxide, and embedded using a graded series of araldite. Embedded leaf pieces were sectioned to a thickness of 5 μm. Sections were dried onto poly-prep slides (Sigma, P-0425) and images captured using a CCD72 camera mounted on a light microscope (Axiophot, Zeiss, Jena, Germany).

For TEM sections of approximately 70 nm were obtained using an RMC MT6000-XL ultramicrotome and a Diatome Ultra45 diamond knife, and collected on 150 hex-mesh copper grids. The sections were stained with saturated aqueous uranyl acetate for 9 minutes and counter-stained with Reynold’s lead citrate for 5 minutes. The air-dried samples were examined on a Hitachi H7500 TEM and images captured using a Gatan US1000 digital camera and digital Micrograph v1.82.366 software.

### Calcium measurements

Plants were grown for 4 weeks in soil, the leaves harvested, and freeze dried. Mineral analysis was conducted using inductively coupled plasma (ICP) atomic emission absorption spectrometry on weighed leaf samples (Soil, Water and Forage Testing Laboratory, Texas A&M University). Each measurement was performed in duplicate on three independently grown sets of plants, the results averaged, and standard error calculated.

### Oxalate measurements

Oxalate determinations were done as previously described [25]. In brief, plants were grown in soil for 4 weeks, the leaves harvested, and freeze-dried. The freeze dried leaf samples were weighed, ground in water, and centrifuged. The supernatant was decanted and analyzed for soluble oxalate levels using the oxalate diagnostic kit (Trinity Biotech, St. Louis, MO). Total oxalate levels were determined by simply omitting the centrifugation step and solubilizing the crystals [25]. Crystals were solubilized by the addition of H^+^-Dowex in dilute acid. The mixture was heated at 60°C for 1 h to dissolve the oxalate crystals. The pH of the mixture was then adjusted (pH 5–7), followed by charcoal filtration and centrifugation. The supernatant was then analyzed for oxalate content according to the manufacturer’s instructions (Trinity Biotech). In brief, the oxalate was oxidized by oxalate oxidase to carbon dioxide and hydrogen peroxide. The generated hydrogen peroxide was then allowed to react with 3-methyl-2-benzothiazolinone hydrazone and 3-(dimethylamino) benzoic acid in the presence of peroxidase to produce an indamine dye that was detected at 590 nm. Standards were prepared from oxalic acid dihydrate (Sigma) and used for both soluble and total oxalate measurements as recommended by the manufacturer. Measurements were performed in duplicate on three independently grown sets of plants, the results averaged, and standard error calculated.

## Results

### Ectopic expression of obc1 in *Arabidopsis*

**Fig 1:**
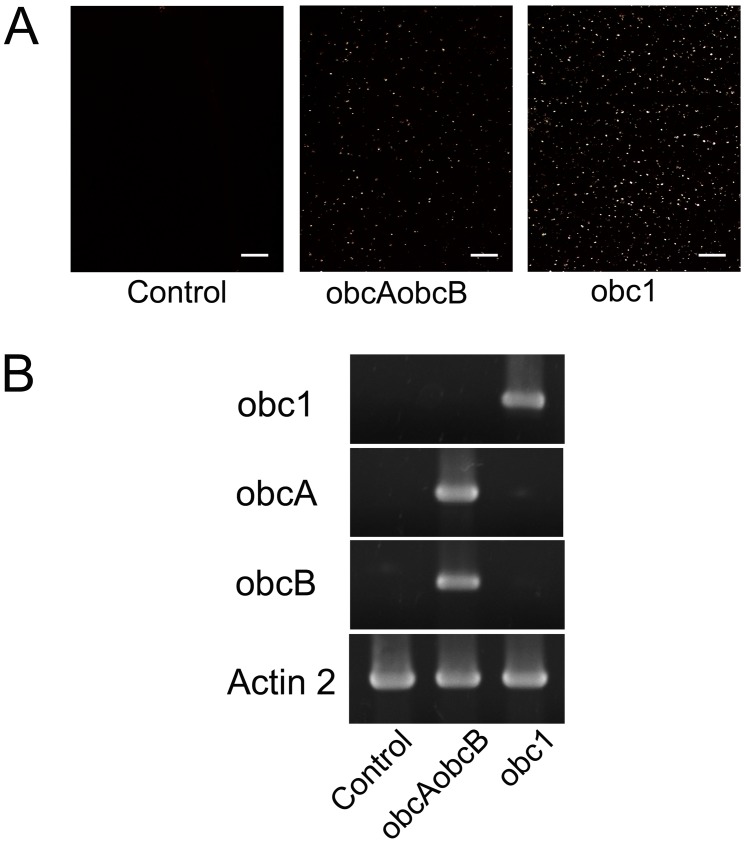
Engineered calcium oxalate crystal formation. (A) A representative whole-mount *Arabidopsis* leaf from control and engineered plant lines. All bars = 100 μm. (B) Ectopic expression of obc genes in *Arabidopsis*. RNA was isolated from leaves of 4 week old plants. Expression of obc1 and actin 2 (control) were assessed by RT-PCR using gene-specific primers and total RNA as template. The produced cDNA fragments were size fractionated by agarose gel electrophoresis.

The obc1 gene, encoding the oxalate biosynthetic enzyme from *B*. *mallei*, was ectopically expressed in *Arabidopsis* using the 35S cauliflower mosaic virus (CaMV) promoter in an effort to produce ample amounts of calcium oxalate crystals. Microscopic inspection of the leaves from the obc1 engineered plants revealed birefringent crystals (Fig 1A). Based on the crystal phenotype more crystals appeared to accumulate in the obc1 expressing plant than in plants expressing the oxalate biosynthetic genes, obcA and obcB from *B*. *glumae* (Fig 1A). To confirm that the observed crystal phenotype was a result of the expression of the obc transgenes, reverse transcription PCR was performed to detect transcript production. As expected only plants expressing the obc transgenes showed crystal accumulation (Fig 1B).

### Localization of calcium oxalate crystal deposition

**Fig 2:**
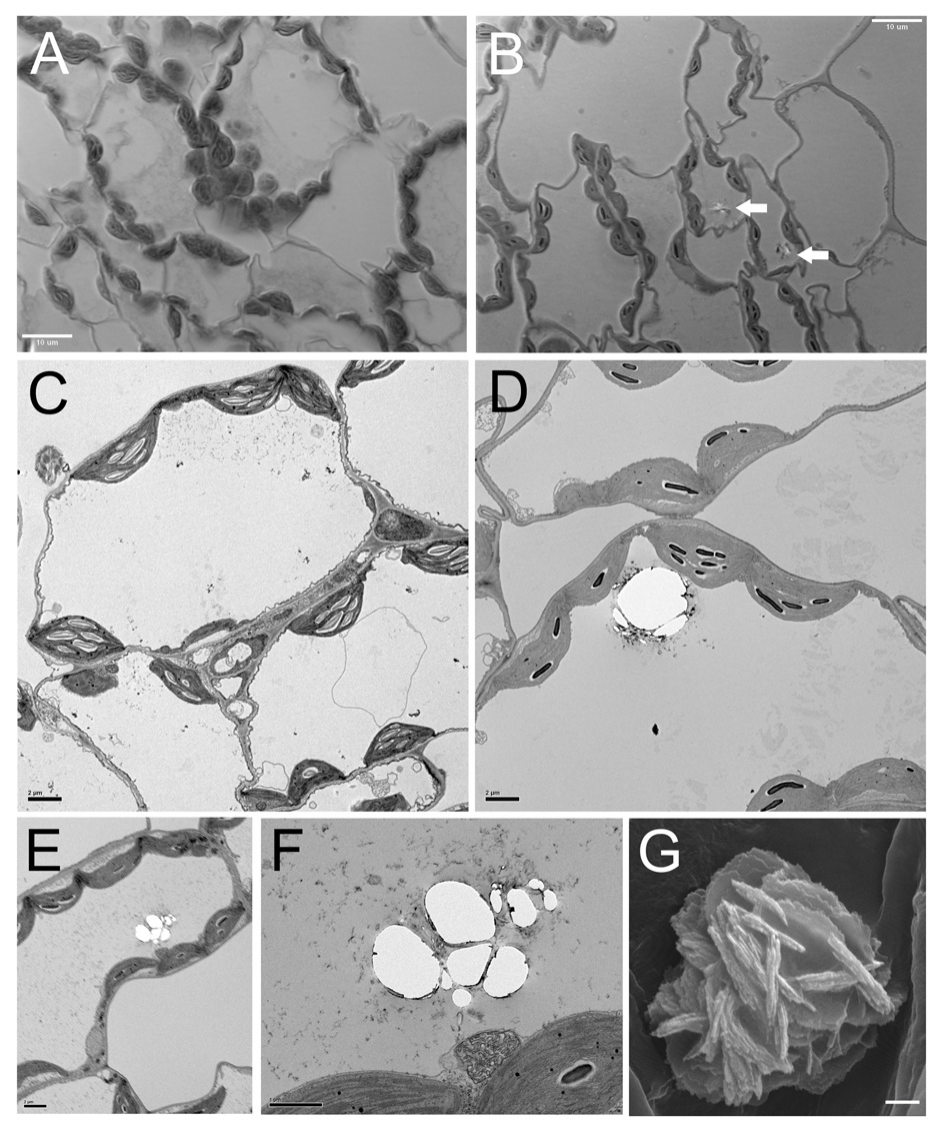
Localization of engineered calcium oxalate crystals. (A) Light micrograph of leaf cross-sections from *Arabidopsis* control (B) Light micrographs of leaf cross-sections from a representative engineered *Arabidopsis* expressing obc1. Bar = 10 μm. (C) TEM of leaf cross-section from *Arabidopsis* control. (D-F) TEM of leaf cross-section from a representative engineered *Arabidopsis* expressing obc1. Bar = 2 μm. (G) SEM of calcium oxalate crystal from engineered *Arabidopsis* expressing obc1. Bar = 1 μm.

To determine if the intracellular location of the crystals in the obc1 plants were in the same intracellular compartment as they are found in naturally occurring crystal forming plants, leaf pieces of the obc1 expressing plants were fixed, embedded in plastic resin, and sectioned. Light micrographs of sections of control (Fig 2A) and transgenic crystal-containing cells suggested that the crystals accumulate within the vacuoles (Fig 2B). This was verified upon inspecting thin sections using transmission electron microscopy (Fig 2C–2F). Crystals were observed within the vacuoles associated with membrane-like structures (Fig 2F). The overall morphology of the engineered crystals appeared to possess a floral bud like appearance (Fig 2G) similar to the druse crystals reported in *M*. *truncatula* [20, 25]. In addition, energy-dispersive X-ray microanalysis confirmed that the engineered oxalate crystals were composed primarily of calcium (S1 Fig).

### Calcium oxalate concentration

**Fig 3:**
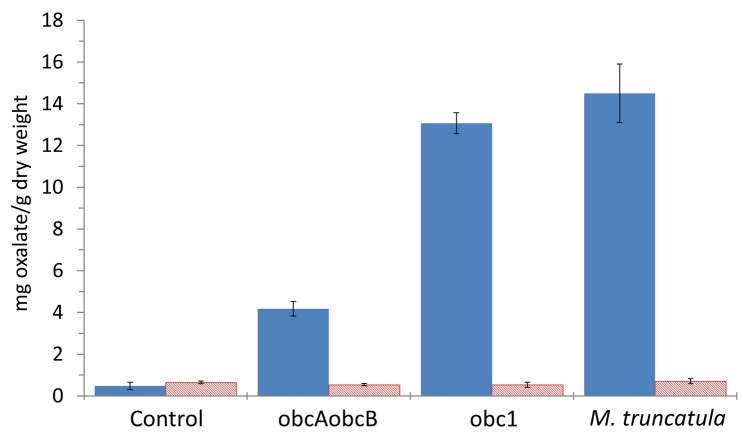
Oxalate concentrations. Leaves were isolated from 4-week old plants, frozen in liquid nitrogen, and freeze-dried. Oxalate measurements were conducted using an enzymatic assay on weighed leaf samples. Values represent averages from three different samplings (mean ± SE). Total oxalate shown as solid bars and soluble oxalate represented by hatched bars.

Total and soluble oxalate measurements were conducted on control and engineered plants to assess whether the observed crystal accumulation correlated with oxalate concentrations (Fig 3). Measurement of control leaves showed low total oxalate concentration of about 0.48 mg g-1 DW. The transgenic lines showed varying amount of oxalate. The highest amount of insoluble oxalate was measured in the obc1 line at approximately 13.07 mg g-1 DW. This amount was similar to the amount measured in calcium oxalate crystal accumulating plants such as *M*. *truncatula* (Fig 3). An intermediate amount of oxalate, roughly 4.17 mg g-1 DW, was measured in the obcAobcB expressing plant (Fig 3). All plants showed similar low levels of soluble oxalate (Fig 3).

**Fig 4:**
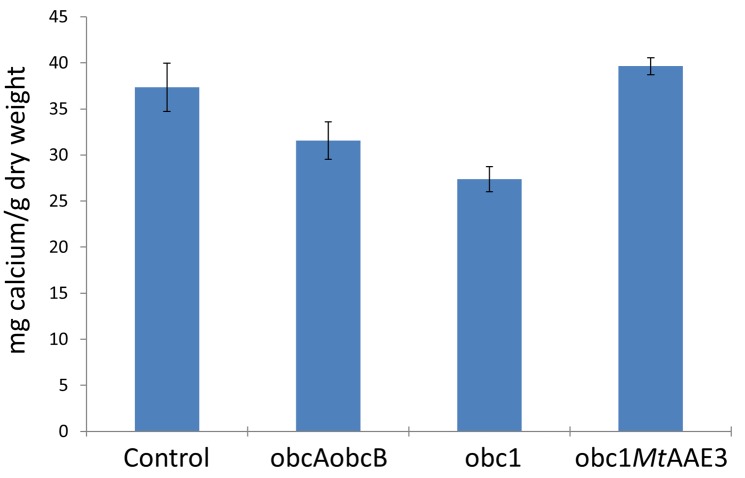
Calcium concentrations. Leaves were isolated from 4-week old plants, frozen in liquid nitrogen, and freeze-dried. Calcium measurements were conducted using inductively coupled plasma atomic emission absorption on weighed leaf samples. Values represent averages of three samplings (mean ± SE).

To assess whether the calcium concentration changed in correlation with the increase in oxalate amount, the mineral was measured using ICP. There appeared to be a general trend of decreasing calcium concentration with increasing oxalate concentrations in the engineered and control plants (Fig 4). This finding coupled with the varying calcium oxalate concentrations measured in the different plant lines indicated that these engineered lines have altered the amount of tissue calcium that was partitioned into the oxalate crystals.

### Effect of engineering calcium oxalate accumulation on plant growth and development

**Fig 5:**
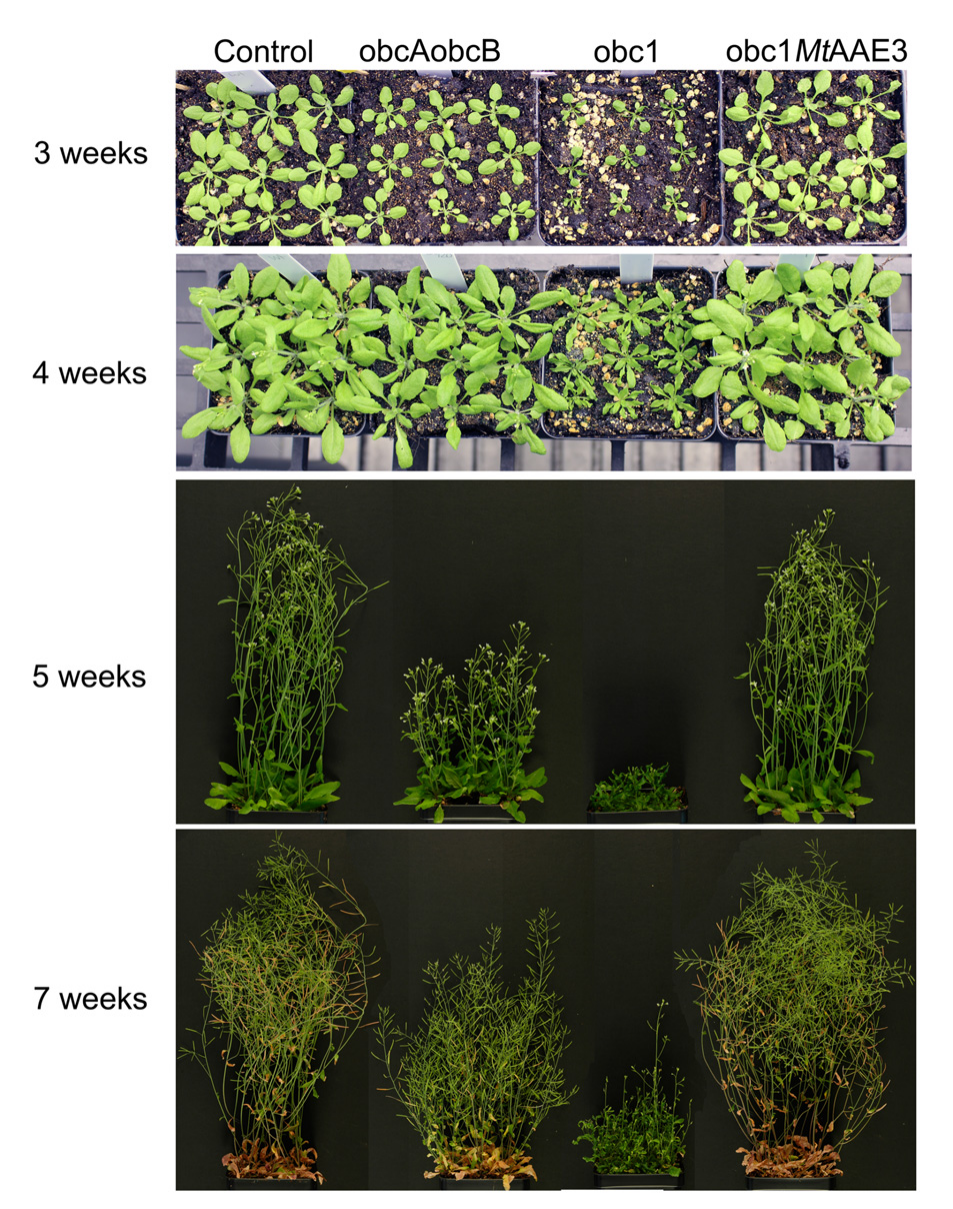
Phenotypes of control and engineered calcium oxalate accumulating *Arabidopsis*. Representative photos of a pot of nine Control, obcAobcB, obc1, or obc1*MtAAE3 Arabidopsis* plants at 3, 4, 5, and 7 weeks of growth and development.

As a step toward determining the effects of engineering calcium oxalate crystal formation in non-crystal forming plants the phenotype of the obc expressing plants were compared to wild-type at different time points during growth and development (Fig 5). The plants engineered to form crystals of calcium oxalate displayed a reduction in plant stature compared to controls with the most severe phenotype observed with the obc1 expressing plants (Fig 5). The obc1 expressing plant also exhibited a more pronounced delay in bolting and seed set (Fig 5).

### Restoration of wild type plant growth and development

**Fig 6:**
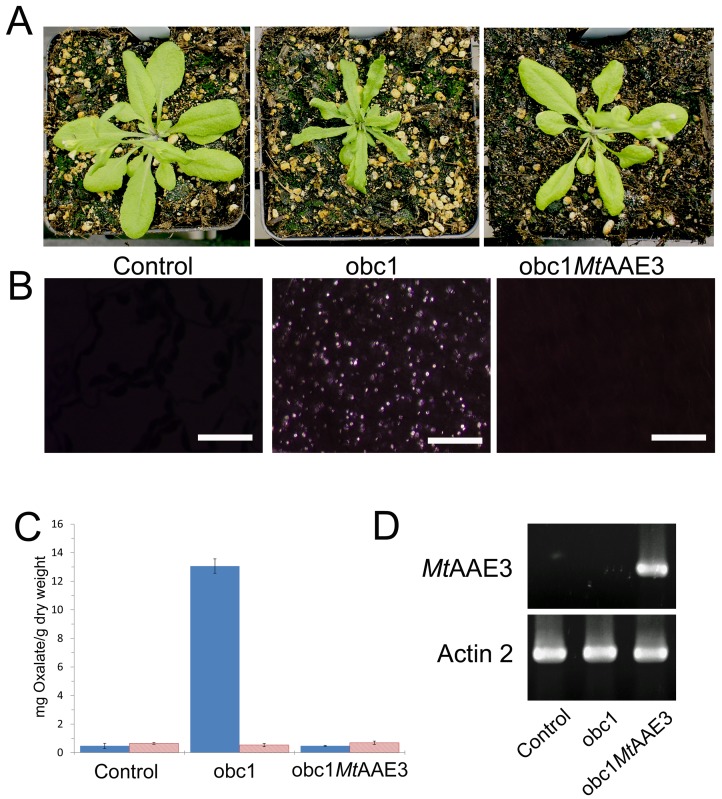
Rescue of control plant phenotypes in obc1 expressing plants. (A) Plant phenotypes (B) Crystal phenotypes (C) Calcium oxalate concentrations. Total oxalate shown as solid bars and soluble oxalate represented by hatched bars. (D) *MtAAE3* expression.

To verify that the observed plant phenotypes were due to an increase in calcium oxalate crystal accumulation rather than a reduction in oxaloacetate or acetyl-CoA (the substrates for the obc1 enzyme), the obc1 expressing plant was retransformed with the *M*. *truncatula* oxalyl-CoA synthetase (MtAAE3). Oxalyl-CoA synthetases were recently shown to convert oxalate to oxalyl-CoA as a first step in the process of oxalate turnover in plants [22] and yeast [23]. The over-expression of the MtAAE3 in the obc1 expressing plant was able to restore the wild-type plant phenotype (Fig 6A) suggesting that the aberrant plant growth phenotype was due to the formation of calcium oxalate crystals rather than a reduction in oxaloacetate and acetyl CoA, the substrates of the obc1 enzyme.

To verify that the observed restoration of phenotype correlated with a reduction in calcium oxalate crystal accumulation leaves from each plant were visually inspected using light microscopy. The view between crossed-polarizers showed a reduction in calcium oxalate crystals (Fig 6B). This was confirmed by oxalate measurements conducted on control and engineered plant lines (Fig 6C). Calcium concentrations were also restored to wildtype levels (Fig 4). Simple supplementation of obc1 plants with an exogenous supply of calcium did not rescue the growth phenotype (S2 Fig).

To confirm that the varying amounts of observed crystals correlated with the expression of the *M*. *truncatula* oxalyl-CoA synthetase transgene reverse transcription PCR was performed (Fig 6D). The results showed that the obc1 expressing plants that also expressed oxalyl-CoA synthetase gene showed a wild type plant phenotype (Fig 6D).

## Discussion

The deterring effects of calcium oxalate crystals against herbivory coupled with the knowledge that these crystals are largely absent from a number of important crop plants raises the possibility of exploiting this trait for enhancing plant protection. As an initial step toward this realization, it was recently shown that *Arabidopsis* could be engineered to form crystals of calcium oxalate [20] through the expression of two genes, obcA and obcB, that together encode the oxalate biosynthetic enzyme from *B glumae* [26]. This study was a major first step in our efforts to develop calcium oxalate crystal formation as a new strategy to improve plant protection. Questions remain; however, regarding the feasibility of this approach. For example, the calcium oxalate concentration reached using the obcAobcB genes was approximately 5 times lower than the amount measured in *M*. *truncatula*, a plant where the crystals have been shown to have an insect deterring function [18]. Can non-crystal accumulating plants such as *Arabidopsis* be engineered to accumulate higher calcium oxalate concentrations similar to *M*. *truncatula*? And if so, would there be any pleiotropic effects on the growth and development of the plant?

This study was aimed toward addressing these questions by first generating a calcium oxalate crystal forming *Arabidopsis* line with higher calcium oxalate concentrations through the expression of a single gene, obc1, from the oxalate-secreting bacterium *B*. *mallei* [21]. Utilization of this single gene expression strategy not only allowed easier transgenic manipulations, but also enabled the achievement of the desired higher concentrations of tissue calcium oxalate crystal accumulation similar to the concentrations measured in *M*. *truncatula*. In addition, obtaining a higher crystal producing plant line allowed a better assessment of the total calcium oxalate accumulating capacity of the plant. The increase in calcium oxalate production did not alter the plants ability to transport the oxalate to the vacuole where crystal formation occurs. Therefore, the capacity of the calcium oxalate crystal formation machinery has not been exceeded leaving open the possibility of achieving higher calcium oxalate tissue concentrations.

A consequence of engineering higher concentrations of tissue calcium oxalate; however, was observed with visible alterations in plant growth and development. With the increased concentration of crystal deposition, the obc1 expressing *Arabidopsis* exhibited a smaller rosette with narrower leaves. Each leaf also had an undulating or wavy appearance. Such a phenotype was less pronounced in the obcAobcB plant which accumulated a lower concentration of calcium oxalate. Thus, the observed alterations in plant growth and development could result from the accumulation of the higher tissue concentrations of calcium oxalate or a depletion of the obc1 substrates, oxaloacetate and acetyl-CoA. The rescue of the wild type plant phenotype through removal of the produced oxalate by overexpression of MtAAE3 suggests that the higher tissue calcium oxalate concentration rather than a depletion of obc1 substrates is responsible for the aberrant phenotypes. Whether the observed phenotypes are a consequence of the oxalate crystals themselves, the repartitioning of tissue calcium in the formation of these crystals, the cell-types accumulating the crystals or a combination of these events remain to be determined. In *M*. *truncatula* the insect deterring crystals are restricted to specialized calcium oxalate accumulating cells called crystal idioblasts. In contrast to other cell-types, crystal idioblasts have been shown to contain unique structural features that have been suggested to aid in the formation of the calcium oxalate crystals (reviewed by [5–8]). Such unique features include extensive ER networks, abundant golgi, specialized plastids, and specialized vacuolar components. In addition, these crystal idioblasts are found surrounding the secondary vascular strands in *M*. *truncatula* [25]. This is in contrast to the spatial pattern of crystal accumulation within the engineered *Arabidopsis* which was generated utilizing the broad cell-type CAMV 35S promoter. This difference in spatial distribution could result in localized calcium deficiencies. Thus, utilizing a cell-specific promoter rather than the CAMV 35S promoter would be a logical next step in trying to alleviate the retarded growth and development phenotype.

An increase in the size of the engineered calcium oxalate crystal may also be required to confer insect resistance. Previously, factors such as crystal size [18, 19] and shape [27] were shown to be important factors in conferring resistance to chewing insects. A study by Park et al., 2009, showed a chewing caterpillar, *Spodoptera exigua*, readily tolerated the application of commercial preparations of small amorphous calcium oxalate crystals in their diet. In addition, the *M truncatula* mutant, *cod4*, which has an increase in calcium oxalate concentrations due to an increase in druse crystal formation within the mesophyll tissue did not show any increase in resistance to chewing insects compared to controls [19]. In both instances, examination of the mandibles isolated from the caterpillars fed the small crystals showed no abrasive wear. Therefore, the size of the crystal appears to be an important deterring factor.

Recently, Konno et al., [27] showed, using isolated raphide crystals of calcium oxalate, that the needle-shape of the crystal was a crucial factor in a synergistic defense function when coupled with an exogenous application of cysteine protease. Raphides alone or cysteine protease alone were found to have only a weak defensive function against the larvae of the Eri silkmoth. Co-supplementation of raphides and protease showed a strong larval growth reducing activity. The substitution of amorphous calcium oxalate crystals for the raphides did not show the same synergistic effect suggesting that a “needle-effect” was facilitating the activity of the protease. Thus, the ability to increase crystal size and/or shape are parameters that may need to be developed before a rational strategy can be designed to utilize calcium oxalate crystal formation to engineer resistance against chewing insects in non-crystal accumulating crop plants.

In summary, this study provides a starting basis for the further optimization of engineering calcium oxalate crystal formation in plants with the future intent of increasing plant protection against herbivory. Successful implementation of such a strategy would provide multiple benefits. One such benefit would be as an alternative to the use of chemicals and other pesticides and another would be as a management strategy to decrease insect resistance to the pesticides currently used in crop production. This of course is dependent on the design of strategies that minimize the negative impacts of engineering crystal formation of plant growth and development. Future studies will be aimed toward addressing these issues.

## Supporting Information

S1 Fig Energy-dispersive X-ray microanalysis. Mineral microanalysis was conducted on crystals isolated from obc1 plants. Leaf tissue was homogenized and the crystals pelleted using a microcentrifuge. An aliquot of the crystal sediment was placed on a stub, allowed to air-dry, and viewed using a Hitachi SU8 230 scanning electron microscope (SEM). The SEM was fitted with a Bruker energy dispersive X-ray analyzer (Baylor College of Medicine). The spectra of elements were obtained by focusing the bean at high magnification on individual crystals and collecting the emitted X-rays.
(TIF)

S2 Fig Calcium supplementation on plant growth. WT and obc1 plants were germinated and grown on plates containing 1X Gamborgs pH 5.8 solidified with 0.8% agar. After 1 week, sets of plants (9) were transferred to pots containing Sunshine Professional Growing Mix. Pots containing the WT and obc1 plants then were divided into 3 groups which were supplemented with 0 mM, 5 mM, or 10 mM CaCl_2_ and grown for an additional 3 weeks. Plant growth was monitored and images recorded weekly.
(TIF)
