# Supplementary figures and images for: An Assessment of Engineered Calcium Oxalate Crystal Formation on Plant Growth and Development as a Step toward Evaluating Its Use to Enhance Plant Defense
Source: PLoS One. 2015 Oct 30;10(10):e0141982. doi: 10.1371/journal.pone.0141982 (PMC4627732; doi:10.1371/journal.pone.0141982)

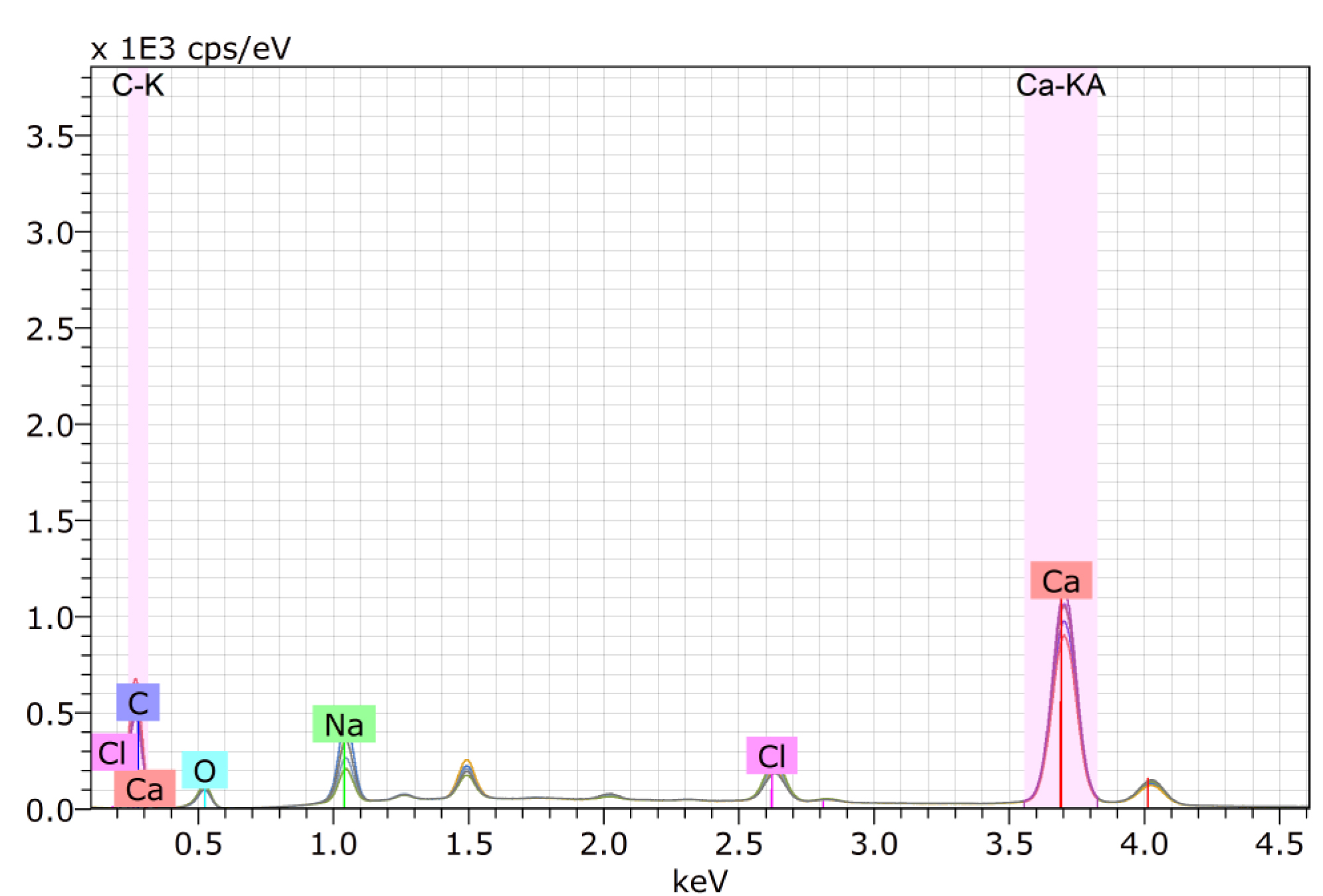

Supplement: S1 Fig — Mineral microanalysis was conducted on crystals isolated from obc1 plants. Leaf tissue was homogenized and the crystals pelleted using a microcentrifuge. An aliquot of the crystal sediment was placed on a stub, allowed to air-dry, and viewed using a Hitachi SU8 230 scanning electron microscope (SEM). The SEM was fitted with a Bruker energy dispersive X-ray analyzer (Baylor College of Medicine). The spectra of elements were obtained by focusing the bean at high magnification on individual crystals and collecting the emitted X-rays. (TIF) [file pone.0141982.s001.tif]

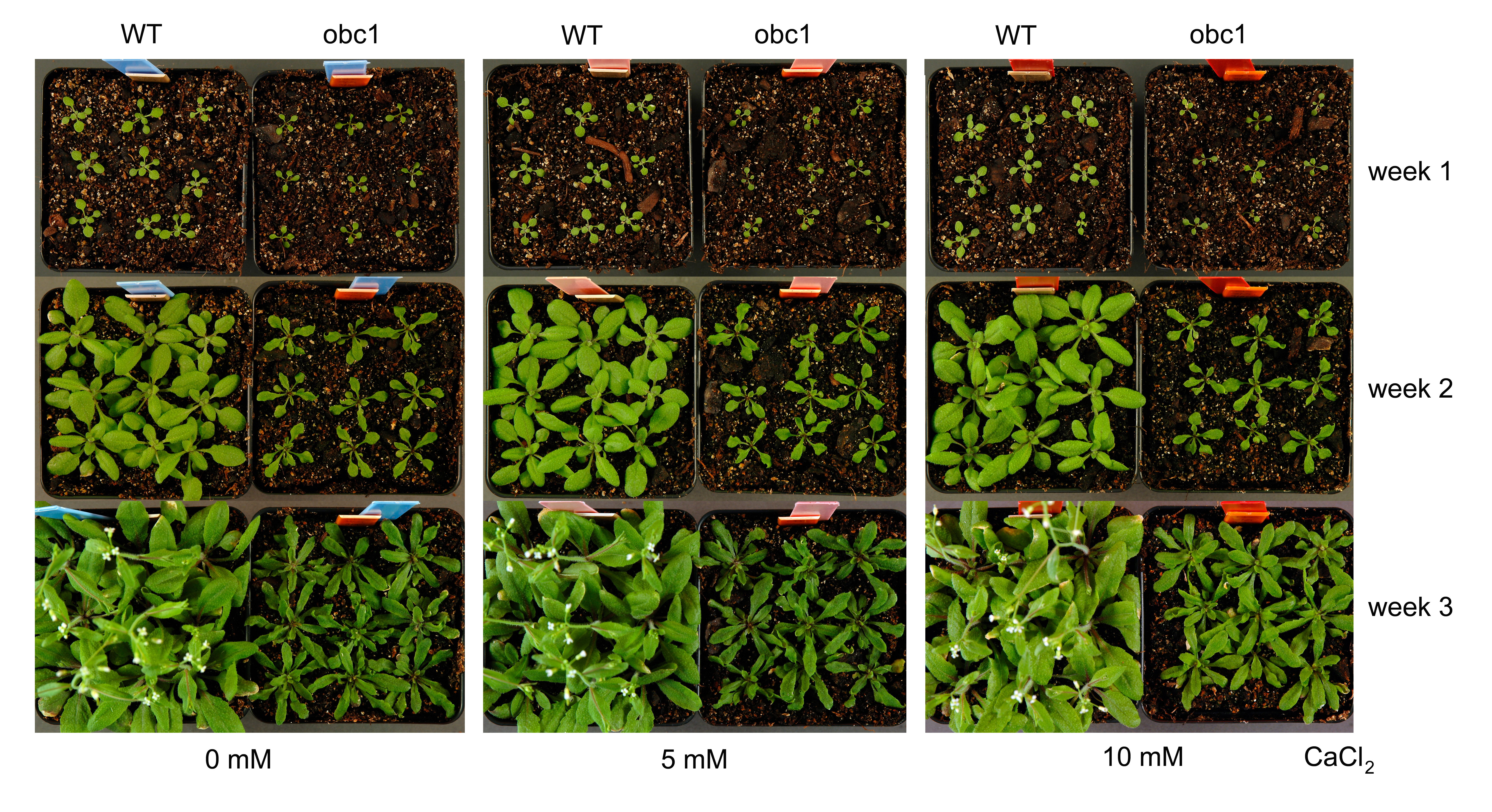

Supplement: S2 Fig — WT and obc1 plants were germinated and grown on plates containing 1X Gamborgs pH 5.8 solidified with 0.8% agar. After 1 week, sets of plants (9) were transferred to pots containing Sunshine Professional Growing Mix. Pots containing the WT and obc1 plants then were divided into 3 groups which were supplemented with 0 mM, 5 mM, or 10 mM CaCl2 and grown for an additional 3 weeks. Plant growth was monitored and images recorded weekly. (TIF) [file pone.0141982.s002.tif]
